# Effects of Degree and Timing of Social Housing on Reversal Learning and Response to Novel Objects in Dairy Calves

**DOI:** 10.1371/journal.pone.0132828

**Published:** 2015-08-14

**Authors:** Rebecca K. Meagher, Rolnei R. Daros, João H. C. Costa, Marina A. G. von Keyserlingk, Maria J. Hötzel, Daniel M. Weary

**Affiliations:** 1 Animal Welfare Program, Faculty of Land and Food Systems, University of British Columbia, Vancouver, British Columbia, Canada; 2 Departamento de Zootecnia e Desenvolvimento Rural, Universidade Federal de Santa Catarina, Florianópolis, Santa Catarina, Brazil; Oregon Health and Science University, UNITED STATES

## Abstract

Rodents and primates deprived of early social contact exhibit deficits in learning and behavioural flexibility. They often also exhibit apparent signs of elevated anxiety, although the relationship between these effects has not been studied. To investigate whether dairy calves are similarly affected, we first compared calves housed in standard individual pens (n = 7) to those housed in a dynamic group with access to their mothers (n = 8). All calves learned to approach the correct stimulus in a visual discrimination task. Only one individually housed calf was able to re-learn the task when the stimuli were reversed, compared to all but one calf from the group. A second experiment investigated whether this effect might be explained by anxiety in individually housed animals interfering with their learning, and tested varying degrees of social contact in addition to the complex group: pair housing beginning early (approximately 6 days old) and late (6 weeks old). Again, fewer individually reared calves learned the reversal task (2 of 10 or 20%) compared to early paired and grouped calves (16 of 21 or 76% of calves). Late paired calves had intermediate success. Individually housed calves were slower to touch novel objects, but the magnitude of the fear response did not correlate with reversal performance. We conclude that individually housed calves have learning deficits, but these deficits were not likely associated with increased anxiety.

## Introduction

Social isolation early in life is known to impair aspects of cognition. In rodents, isolation after weaning causes deficits in reversal learning and novel object recognition, along with a host of other changes including increased anxiety-like behaviour, sensation-seeking and hyperactivity in novel environments (reviewed by [1]). Research on varying degrees of social deprivation in primates from birth reports similar effects on learning and anxiety, along with abnormal repetitive behaviours (reviewed by [2,3]), as do studies of humans raised in institutions where very little social contact was provided (e.g. [4,5]). Recent evidence suggests that social isolation during development can likewise impair cognition in cattle. Compared to pair-housed calves, those housed individually from birth made more mistakes during a reversal-learning task and showed evidence of impaired object recognition [6].

One potential explanation for the cognitive and emotional difficulties observed after early life social deprivation is that a sensitive period in brain development affects later flexibility and adaptive capacity. Previous work has shown that social deprivation can influence neural development and brain function as well as behaviour (e.g. primates: [7,8]; piglets: [9,10]; humans: [4,11]). The emotional effects may also contribute to the cognitive ones: anxiety or stress can impair learning (reviewed by [12]: animals], [13]: humans). If the individually reared animals perceive the testing situation as stressful, this might contribute to poor performance (cf. [14]). To our knowledge this connection has not yet been investigated in the case of early isolation.

Dairy calves provide a good model for investigating these effects, since individual housing of calves from birth until weaning is standard practice on farms. This practice was adopted because it was believed to reduce disease transmission and improve control of feeding and ease of management [15]. Unfortunately, it likely reduces calf welfare (i.e. their quality of life and psychological well-being) in a variety of ways. Most obviously, social behaviours are restricted [16–18]; these behaviours are thought to be important because cattle are naturally social [19] and because cattle show signs of fear and distress when separated from their companions (e.g. [20,21]). Individually housed calves exhibit more anxiety-related behaviour in open field tests and novel social situations than do paired or group-housed calves [22,23], and are slower to eat novel feed [24]. Individually reared calves also experience difficulties adjusting to the group housing systems commonly employed for older cattle. For example, these calves are slower to start feeding when introduced into a new pen and therefore gain less weight in the days after grouping [25]. Some research also shows that individually reared calves are more reactive to human handling [26]; together with their response to novel situations, this suggests individual housing may increase reactivity and reduce performance in tasks where a human trainer is present. However, individually housed calves in some studies approach handlers more readily than do socially reared calves [26–28], perhaps because they are more likely to perceive humans as social companions.

The aim of the current study was to test the effects of individual housing versus different degrees of social housing on discrimination and reversal learning in dairy calves. We predicted that socially housed calves would perform better in a reversal-learning task than would individually reared calves. We first compared individually housed calves with those reared in a complex social group. In a second experiment, we added treatments testing whether lower levels of social contact were sufficient to avoid learning impairments. If anxiety mediates the deprivation-induced learning deficit, we expected that increased fear responses in novel object and handling tests would be associated with an increased number of sessions required to learn the reversal task. If the impairment was due to differences in general neural development, we expected that the degree of impairment would not relate to fear behaviour in either test and that the impairment would not be eliminated by later social contact.

## Experiment 1

### Methods

#### Ethics statement

Both experiments were performed at the University of British Columbia (UBC) Dairy Education and Research Centre (Agassiz, BC, Canada), and animals were managed according to its normal practices following the experiment. Animals were cared for according to the guidelines of the Canadian Council on Animal Care (2009) and the National Farm Animal Care Council (NFAAC, 2009). The research was approved by the UBC Animal Care Committee (Protocol A12-0337).

#### Treatments

Fifteen Holstein calves were randomly assigned at birth to Individual (n = 7) or Group housing in a complex environment (n = 8). Individual calves were separated from the dam within 6 h of birth and housed in sawdust-bedded single pens (1.2 × 2m), separated by plastic walls that allowed auditory but not visual contact with neighbouring calves; plywood barriers approximately 1 m in front of the pen blocked their view of calves in the opposite row of pens. Calves assigned to the group housing treatment were kept with their dams in the calving pen (a 4 x 4 m individual pen bedded with straw covering sand, to which cows were moved when they showed signs of parturition onset) for approximately 72 h; the cow and calf were then moved together to the social pen. Cow-calf pairs in this social pen were managed as a dynamic group, with animals added and removed as they reached the appropriate ages; a minimum group size of four calves and four cows was maintained, with a maximum of ten calves (median six across both experiments). Calves stayed in a sawdust-bedded creep area (3.5 x 12.3 m) with fence-line contact with their mothers during the day, and from 19.00 h to 07.00 h the calves had free access to their mothers’ pen (a 9.5 x 12.3 m free-stall pen with deep-bedded sand; see [24] for an illustration of the pen). Cows were fitted with udder nets (Large Mesh Udder Support, Franksville Specialty Company, Phillips, WI) that prevented suckling. These treatments were part of a broader project investigating effects of maternal and other social contact on calves’ behavioural development and welfare [24,29], and the high level of social complexity involved in the Group treatment provided an ideal opportunity for detecting effects on learning abilities. All calves were bottle-fed pasteurized whole milk twice a day; 8 L/d was provided for the first 28d of age, and 6 L/d until gradual weaning from 54 to 56d. Calves had ad libitum access to grain, total mixed ration (mixture of grain, silage and hay), and water throughout the experiment. Calves were weighed and health checks were performed once per week.

#### Training and initial discrimination

Training sessions were conducted twice daily in a training pen (1.2 x 2m) at 07:00 and 16:30, starting when calves reached 7 ± 2 d of age. The full daily milk allowance was obtained in these sessions, but if calves did not finish their milk during a session, the remainder was offered at the end. Calves were trained in a visual discrimination task using a go/no-go paradigm (as in [29]). Stimuli were screens of two colours (red and white) displayed on a monitor, controlled using a computer placed outside of the pen. The colours assigned as positive or negative were alternated between calves in each treatment. “Go” responses (approaching within 20 cm of the monitor) in response to the positive colour were rewarded with milk delivered by bottle. Correct responses to this stimulus were signalled using a clicker until calves were reliably performing the response correctly. “Go” responses following the negative colour were punished with a whistle and 1-min timeout during which they were not given any stimuli or able to obtain milk.

Calves were initially trained in 20-trial sessions including only positive screens until they reached a criterion of over 90% correct response (i.e. when the calves approached the screen by themselves and came back to the reward). In the discrimination phase, negative stimuli were added gradually, increasing from two to six trials per session over three sessions, with the number of positive stimuli remaining constant at 20. Session duration varied with calf performance, lasting as long as necessary for the calf to respond to this number of screens. Negative stimuli were shown for 4 s each. Sessions never had more than six negative stimuli. Training continued until calves reached the learning criterion of 100% of correct responses for negative stimuli (i.e. avoid approaching all six negative stimuli) and 90% correct responses for positive stimuli over two consecutive sessions. The number of negative screens was then gradually increased to 20, after which the reinforcement rate was reduced to 50%. Calves needed to perform at a minimum of 80% correct responses for each stimulus on their last two sessions before continuing to the reversal phase.

#### Reversal learning task

At approximately 45 d of age, after calves had met the learning criterion, the training stimuli were reversed; i.e. the positive stimuli became negative and vice-versa. This phase continued until calves again met the criterion of responding correctly to 100% of negative screens and 90% of positive screens, with a maximum of 13 potential sessions before weaning. As in the initial discrimination training, the reversal learning sessions began with only positive stimuli, and negative stimuli were gradually introduced in sessions 2 to 4. Positive stimuli were shown for up to 20 s in the early sessions of this phase to encourage faster learning, as they had been during the phase of learning to go to the screen in the initial discrimination; after session 4, positive and negative stimuli were shown for a maximum of 4 s, as in the discrimination phase.

#### Statistical analysis

The effect of treatment on the number of sessions to achieve the learning criterion in the discrimination phase was tested using a t-test. Fisher’s exact test was used to compare treatments for the proportion of animals that reached criterion in the reversal task, since some animals never reached the learning criterion. Data files are available at http://hdl.handle.net/11272/10178.

### Results

**Fig 1:**
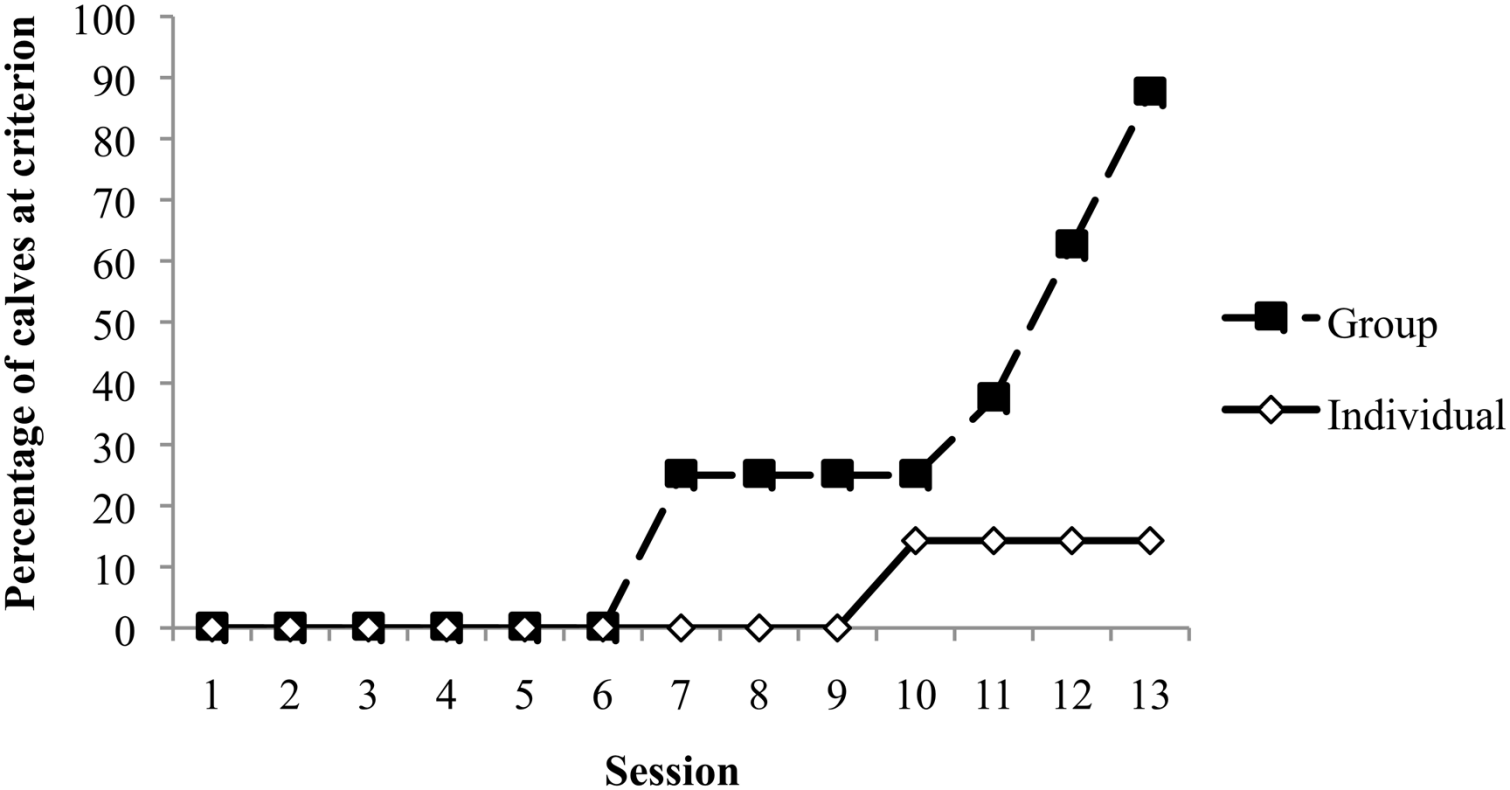
Percentage of calves meeting the learning criterion by treatment over the course of training in the reversal learning task, in Experiment 1. Fewer individual calves successfully learned the reversed colour discrimination by the end of training. n = 7 individual, 8 group calves.

During the discrimination phase, there was no difference between treatments in learning speed (t_13_ = 1.49, *p* = 0.16); group-housed calves reached the learning criterion after an average of 8.9 ± 0.9 training sessions (mean ± SE) versus 10.6 ± 0.6 sessions for Individual calves. In the reversal phase, seven of the eight Group calves were able to reach the learning criterion, requiring on average ± SD 10.3 ± 2.4 sessions; the one remaining calf made only two errors during the twelfth session and none in the thirteenth and final session (see Fig 1). Calves in the Individual treatment were less likely to learn the task (Fisher-exact test; *p* = 0.01); only one out of seven calves reached the criterion, requiring ten sessions. Errors were predominantly in avoiding the negative screen: three calves in this treatment incorrectly approached every negative screen throughout reversal training, while all reached criterion for approaching the positive screen in five sessions or fewer.

## Experiment 2

### Methods

#### Subjects, housing and treatments

A total of 44 Holstein bull calves were assigned to one of four treatments: Individual housing (n = 10), Early Pair (n = 12), Late Pair (n = 12) or Group housing with access to the dam (n = 10). Assignment was random within blocks of four calves, within the constraint that calves closest in age were assigned to pair treatments. Individual and Group calves were housed as in Experiment 1. Nursing was again prevented in the group pen using an udder net. However, two calves still learned to nurse and were thus excluded from the analysis, as extra milk may have interfered with their motivation during training sessions. Early Pair calves were housed individually until they were paired at 6 ± 3 d old by having the barrier to the neighbouring pen removed to create a double pen. Late Pair calves were individually housed until 43 ± 3 d, 6 to 8 d before reversal learning began. Feeding and health were managed as described in Experiment 1.

#### Training and reversal learning

Training for the discrimination task followed the same procedure as in Experiment 1. Reversal learning tests began at approximately 49 d of age. Learning speed was assessed using the number of sessions until the calves achieved the criterion of two consecutive sessions at 100% correct negative responses and at least 90% correct positive responses, at a ratio of 20:6 positive to negative screens. Reversal training continued for a maximum of 22 sessions (two times the median required for the socially housed calves to learn during Experiment 1).

#### Responses to humans and novel objects

Anxiety was inferred using two tests: response to handling during a routine procedure and a novel object test. These tests were conducted on consecutive days before the start of reversal training (approximately 41 d of age and before the Late Pair calves were paired).

Response to human handlers was assessed during weekly weighing, following the procedure of Duve and colleagues [26]. Latency to approach the handler (to a maximum of 90 s) was assessed; in addition, whether the calf retreated from the handler was recorded, and the difficulty in getting the calf onto the scale was scored as 1 (guided gently onto scale), 2 (some pushing required), 3 (one person pushing with maximum force), or 4 (two people required). Responses to this test might be affected by a general increase in anxiety. Moreover, fear of humans was of specific interest because it could have affect the training and cognitive testing in this and previous experiments, which involved close proximity to human handlers.

Novel object tests took place in the training pen. The object was a brightly coloured ball lowered into the pen (at the front and centre of the pen, unless that would necessitate contact with the calf) with a length of twine after 2 min of habituation to the pen. We recorded whether the calf made contact with the object and the latency to do so, along with the number of retreats (stepping away while oriented to the object) or startle responses (a sudden movement of the head or body without taking a step) as an additional indicator of fear. Tests lasted 10 min.

#### Statistical analyses

Fisher’s exact test was used to compare treatments for the proportion of calves able to learn the reversal task in the number of sessions allowed. A General Linear Model (GLM) was used to compare the number of sessions taken to reach criterion for both the initial discrimination phase and the reversal between treatments. If a calf did not reach criterion in the reversal task, a maximum value of 24 was assigned (the number actually completed plus the two additional sessions that would be the minimum required to meet criterion after that). This analysis was also repeated using only calves that were successful in the task. These models controlled for the colour initially assigned as the positive stimulus, and cohort. Normality of residuals for these GLMs was confirmed using Shapiro-Wilk tests. Contrast statements were used to compare Group and Early Pair calves to test for effects of the complexity of the social environment; when no difference was found, these two treatments were combined (then referred to as ‘Early Social’) for comparisons with Individual and Late Pair treatments to test effects of timing of social contact.

For the analyses of responses to humans and novel objects, Late Pair calves were included in the Individual treatment because their data were collected while they were still housed individually. Latency and retreat/startle data from the novel object tests were non-normal according to Shapiro-Wilk tests; treatment differences were analyzed using the Wilcoxon test. Latencies in the handling tests were both non-normal and showed heterogeneity of variance; values were therefore rank-transformed and treatments compared using Welch’s t-tests (following [30]). Due to ceiling effects, with many calves not making contact within the time given, Fisher’s exact tests were also used to compare the proportion of calves not making contact between treatments. Based on barn layout, the direction in which calves had to move to approach the handler relative to the direction they regularly walked to the training pen varied. Since this variable affected both mean latency and variance, and was not balanced across treatments (as Group calves never had to go away from the training pen), all calves for which the handler stood in the opposite direction of the test pen were excluded from these analyses. To test whether behaviour indicative of fear in the response tests was related to reversal learning success, Fisher’s exact tests were used to look at patterns of learning success or failure between calves who made contact with the object or person and those that did not. Where possible, Wilcoxon tests were also used to compare latencies between calves that learned and those that did not among those that did make contact.

### Results

#### Housing effects on learning

All calves completed the discrimination task and there were no differences between treatments in the number of sessions taken to reach criterion (mean ± SE for Individual, Early Pair and Group: 8.8 ± 1.6, 11.9 ± 1.9, and 10.1 ± 2.0; F_2,36_ = 1.29, *p* = 0.288; contrast of Individual vs. all social F_1,36_ = 1.75, *p* = 0.195).

**Fig 2:**
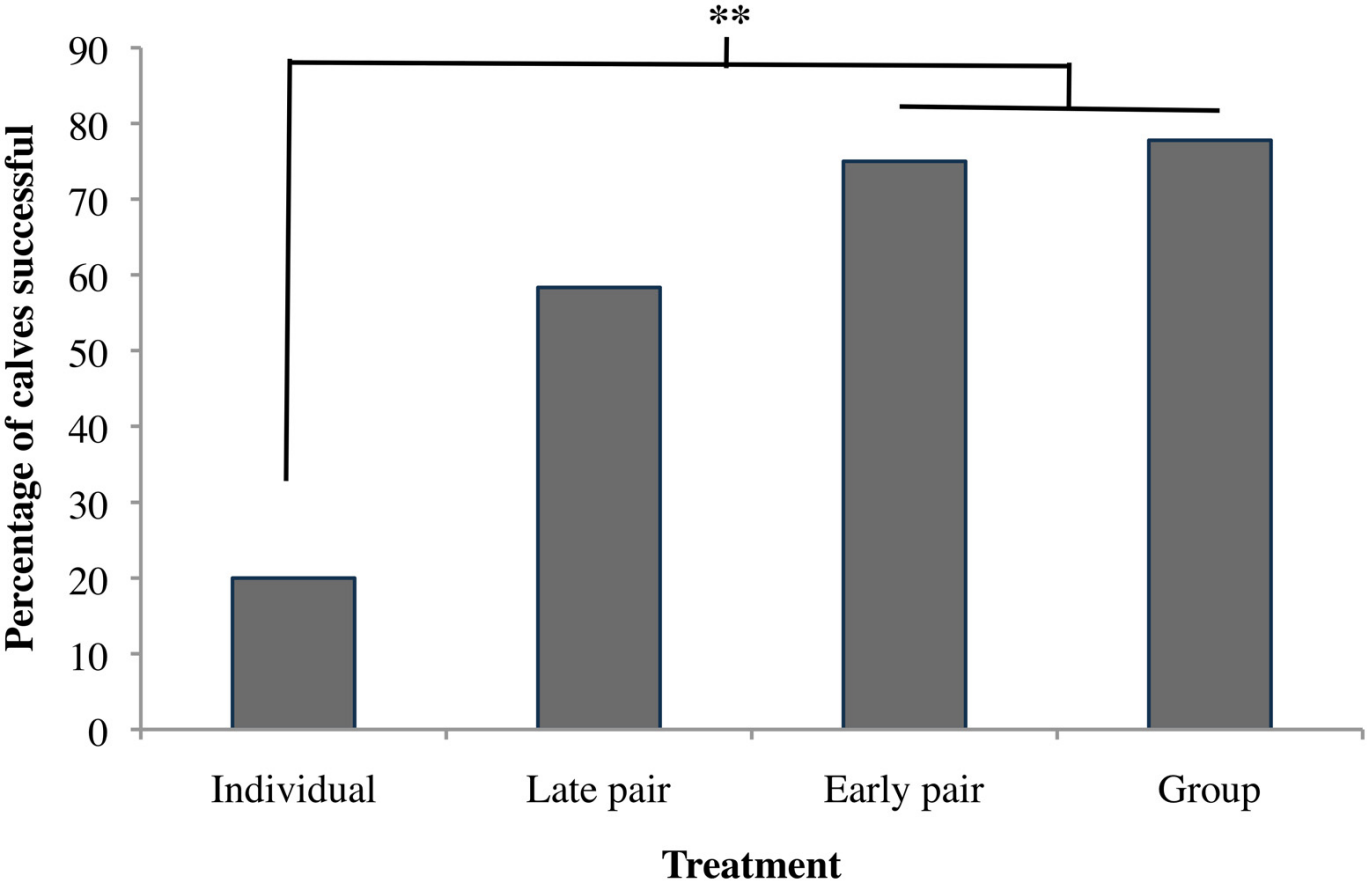
Percentage of calves able to reach criterion in the reversal learning task was higher in calves socially housed in early life than in those housed individually in Experiment 2. ** indicates a significant difference at P<0.05. n = 10 individual, 12 late pair, 12 early pair, 9 group.

Group and Early Pair calves did not differ in the likelihood of learning the reversal task (7 of 9 vs. 9 of 12 calves; *p* = 0.882; Fig 2), and were therefore pooled as the “Early Social” treatments. A higher proportion of these Early Social calves successfully reached criterion than did Individual calves (16 of 21 calves vs. 2 of 10; Fisher’s exact *p* = 0.006). Late Pair calves had intermediate success (7 of 12 calves), not differing from Early Social calves, but tending to be higher than that for Individual calves (*p* = 0.099).

**Fig 3:**
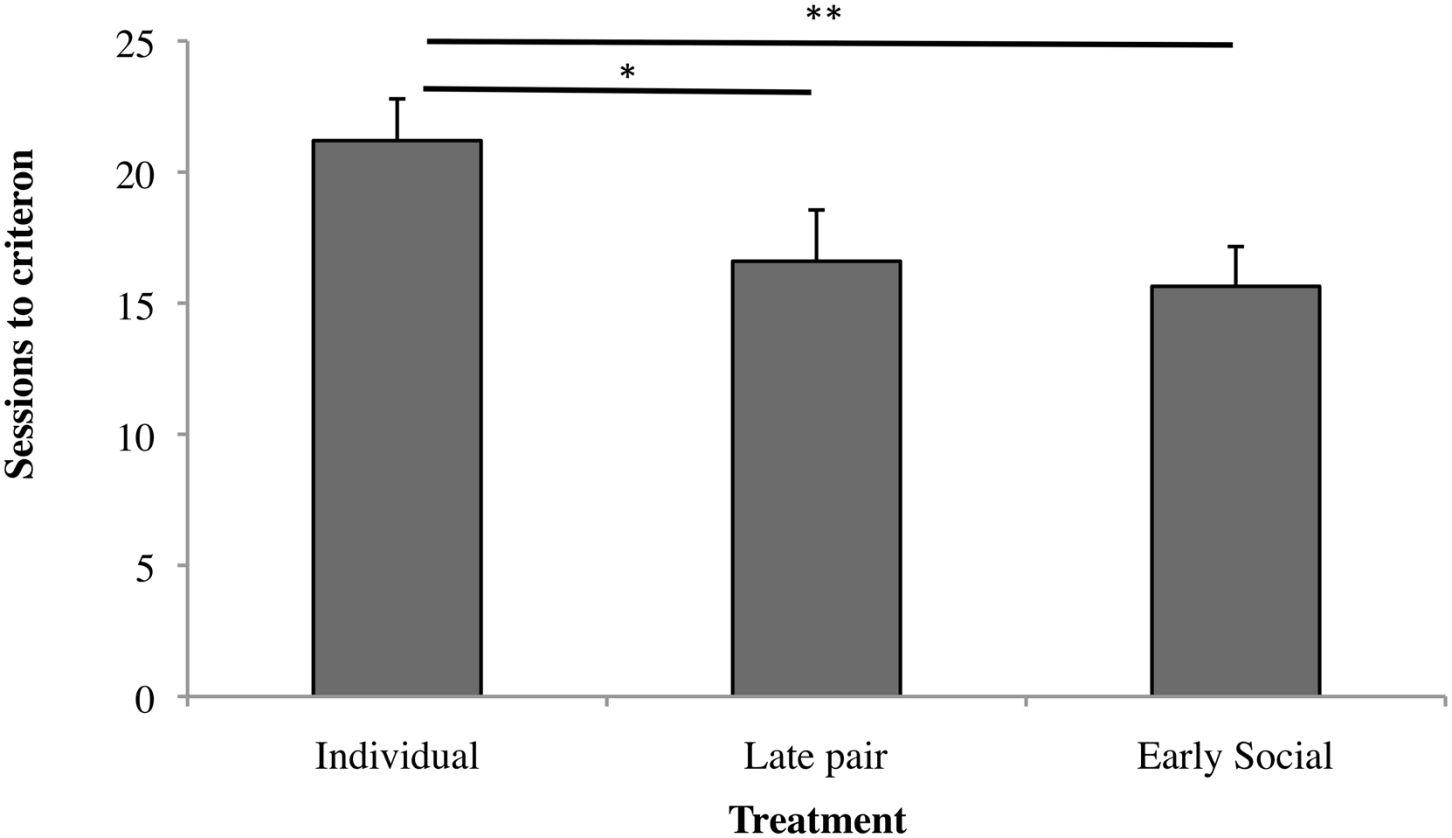
Number of sessions taken to reach criterion in the reversal task in Experiment 2. Values are means + SE (maximum value of 24 assigned to calves who did not learn). Sample sizes as in Fig 2. Early social = calves that were pair- or group-housed since early life (Early Pair + Group). * indicates a statistical tendency (0.05<p<0.10); ** indicates a significant difference (p<0.05).

Fig 3 shows the effect of treatment on the number of sessions needed to reach the learning criterion in the reversal task. Group and Early Pair calves again did not differ (F_1,37_ = 0.02, *p* = 0.892) and were pooled as “Early Social”. Individual calves took longer to learn than did the Early Social calves (F_1,37_ = 6.24, *p* = 0.017). Individual calves also tended to take more sessions than Late Pair calves did (F_1,37_ = 3.24, *p* = 0.080). This effect was driven by calves that failed to learn, with the average number of sessions for successful calves being similar across treatments (least squares means ± SE: Individual 10.0 ± 2.6, Late Pair 13.4 ± 1.9, Early Pair 13.1 ± 1.9, Group 13.9 ± 1.5; F_1,19_≤1.44, *p*≥0.245).

#### Housing effects on responses to humans and novel objects

**Fig 4:**
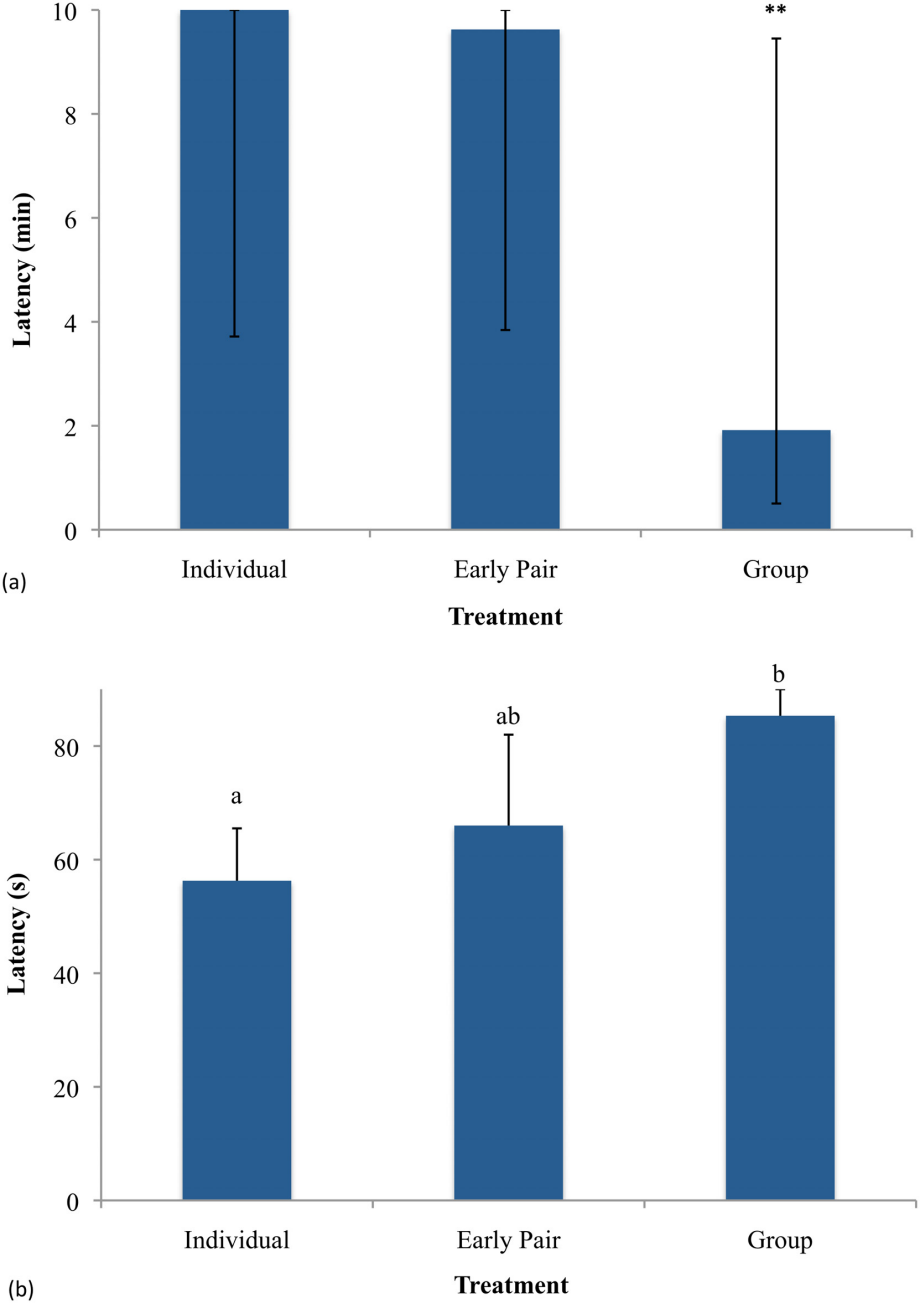
Response of calves to anxiety tests at approx. 40–41 d of age. n = 22 Individual, 13 Early Pair, 9 Group. **(a) Latency to touch a novel object was lowest in group-housed calves at approx. 40 d of age**. Values are medians with interquartile ranges (maximum value of 10 min assigned to calves who did not make contact). ** indicates a treatment that significantly differs from the others at P<0.05. (**b) Latency of calves to touch a familiar human in a handling test was higher in group-housed than in individually housed calves at approximately 41 d of age**. Values are means + SE (maximum value of 90 s assigned to calves who did not make contact). Treatments that do not have the same letter differ from each other at P<0.05.

Latency to make contact with the novel object was longer for Individual (including the as yet unpaired Late Pair calves) and Early Pair calves than for Group calves (Z = -2.33, *p* = 0.020, and Z = 2.40, *p* = 0.017, respectively). Individual and Early Pair calves did not differ (Z = 0.0, *p* = 1.00; Fig 4a). The higher latencies in Individual and Early Pair calves reflect the many calves that never made contact with the object and were therefore assigned the maximum latency. This happened in almost half of all tests (18 of 40 calves), but was numerically more common in Individual versus Group calves (6 of 14 vs. 2 of 10; Fisher’s exact *p* = 0.119). Among calves that did make contact with the object, latency to do so was higher for Pair than Group calves (median 293 vs. 75 s, Z = 2.26, *p* = 0.024) with no differences between other pairs of relevant treatments. There were no differences between treatments for number of retreats or startle responses during this test (Z≤0.762, *p*≥0.445).

Latency to touch a human handler was longer in Group calves than in Individual calves (F_1,18_ = 5.82, *p* = 0.027; Fig 4b); Early Pair calves did not differ from either of these treatments (vs. Group, F_1,9_ = 3.35, *p* = 0.102; vs. Individual, F_1,26_ = 2.41, *p* = 0.133). As in the novel object tests, many calves (17 of 28) never made contact. This was more common in Group calves than in Individuals (8 of 9 vs. 6 of 14; Fisher’s exact *p* = 0.037). Early Pair calves again did not differ from either treatment, with three of five calves not touching the person (*p*≥0.506). Too few calves of some treatments made contact for latencies to be compared among them.

#### Relationship between fearful behaviour towards humans and novel objects and learning

Of the 18 calves that never touched the novel object, 10 (56%) failed to learn the reversal task, whereas just 6 of the 22 calves (23%) that did approach and touch the coloured ball failed to learn (χ^2^ = 3.30, *p* = 0.069). Excluding the calves that did not make contact, latency to make contact in the novel object test did not differ between calves that were successful in the reversal task and those that were not (median 168 vs. 114 s, Z = -074, *p* = 0.460). There was no difference in the learning success of calves that touched a human handler compared with those that did not (5 of 14 calves vs. 13 of 26; *p* = 0.696).

### Discussion

These results show that calves housed with social companions from an early age perform better in certain learning tasks than do those housed individually. The majority of individually housed calves could not learn the reversal task, even when provided twice as many sessions to do so as the average socially housed calf required. This impairment in reversal learning indicates a lack of behavioural flexibility, and thus a reduced ability to respond appropriately to changing environments [31,32]. Experiment 2 investigated the effects of amount of social contact (number of companions) and timing of the social contact (beginning at 6 days or 6 weeks of age). The learning deficits were most apparent in individually housed calves; having even one social companion improved reversal-learning performance. Calves paired after 6 weeks of age showed numerically (but not statistically) poorer success rates in the reversal-learning task compared to calves with social contact from very early in life (Early Pair and Group), and consequently tended to require more sessions to learn the task on average. The intermediate performance of these Late Pairs suggests that if a sensitive period for developing these abilities exists, as it does in rodents (reviewed by [1]), it likely extends beyond 6 weeks of age. The sensitive period identified in rats falls somewhat later in their developmental period [33], so further work looking at a broader range of ages may be justified. However, results of one recent study on other aspects of behavioural development in calves suggest that the sensitive period for social contact ends before 6 weeks of age [34].

A second aim of the current experiment was to test if social housing influences anxiety, and if differences in anxiety or fear of humans help account for the learning difficulties. The results of the relevant behavioural tests used are in agreement with past studies using various tests to infer anxiety in both calves and rodents [1,23,26]: animals reared in social deprivation exhibited more fear of novelty, as indicated by increased latency to approach novel objects, but not more fear of humans. The latter result is not surprising, since fear of humans often does not correlate with fear of other stimuli (e.g. [35]). Unlike previous studies [16,23], however, we did not find reduced fear in Early Pair calves. This lack of effect may be due to lower sensitivity of the measures (latency to approach is prone to ceiling effects) and smaller sample sizes in the current study, or differences in the tests employed (novel object vs. open field). The training and cognitive testing beginning as early as one week of age also provided the Individual calves with high levels of contact with humans and a form of enrichment; this may have influenced their anxiety levels compared to those in similar housing in other studies, reducing any difference from socially housed calves. It is unsurprising that the Group treatment would have a stronger effect on fear of novelty than did pair housing; the social and physical complexity of the Group environment was greater, exposing the calves to greater levels of stimulation and much more variability, especially because the dynamic system meant that individuals were regularly being added and removed during the study period. In addition, maternal behaviours such as licking and grooming have important effects on later stress reactivity in other species [36,37].

There are, of course, limitations to the tests used. The tests are assumed to reflect underlying anxiety or fearfulness because such a state would increase the animal’s tendency to interpret the test stimulus (person or object) as a potential threat and thus exhibit fear (see e.g. [38]); contact with a novel object is, accordingly, increased by treatment with an anxiolytic drug ([39]). However, measures such as latency and duration of contact with an object are imperfect indicators because they are likely also affected by temporary states such as hunger or motivation to rest (see e.g. [40,41]) and individuals may differ in their behavioural expressions of fear [42]. Future experiments could reduce variation caused by these factors by averaging fear measures across repeated tests and using multiple measures to assess anxiety. For the present, the similarity between the current results and those of previous studies using different tests of fearfulness support our interpretation of higher latencies as reflecting fear of novelty. One potential explanation for socially deprived animals behaving more cautiously in novel situations than socially reared ones is that they have less experience with changing stimuli in their environments and so the situation is, in effect, more novel to them (cf. [3]); this does not change the interpretation regarding Individual calves’ fear of novelty, but might mean they are no more anxious in a familiar, unchanging environment.

The increased fear of novelty in Individual calves allowed for the possibility that greater anxiety could explain their poor learning performance. Such effects have been hinted at in cattle: Boissy and Le Neindre [20] found that in one breed, the presence of conspecifics during training on a simple learning task improved learning speed compared to those trained in social isolation. This was attributed to social buffering of stress allowing them to adapt to the situation more quickly. Beef calves and lambs have difficulties learning when experiencing chronic stress [43,44]. Our results are consistent with this hypothesis; calves who never approached the object (potentially the most fearful individuals) tended to be less likely to learn the reversal task. However, the pattern of responses to novel objects across treatments in our study did not align with the pattern of learning difficulties. Early Pair calves, while achieving similar levels of success to those of Group calves in the reversal task, did not show reductions in fear of the novel object. Stress related to human presence during testing can be ruled out as a possible cause of failure to learn in Individual calves, as these calves did not exhibit greater fear of humans. Instead, as in previous studies [26,28], they seemed more interested in human contact. Thus, the current evidence suggests that differences in fear in the testing situation cannot fully explain the cognitive effects of early social deprivation.

How, then, does social deprivation influence cognition? Early hypothesized explanations included the failure of specific neural systems to develop because the animal lacked opportunities to perform certain behaviours [3] or, more generally, the animal lacked experiences that required adaptation and thus did not develop adaptive capabilities [45]. Early social deprivation does result in neurological differences from non-deprived individuals, but the specific features of social experience that are critical to these changes, or their biological mechanisms, are largely still unknown [1]. Cognitive development, and particularly developing the behavioural flexibility to adapt to new situations, is widely believed to depend on exposure to a variable environment in early life [45,46]. Most discussions of the impact of the social environment on cognition and the brain accordingly focus on companions providing variability in the environment and associated cognitive challenges [47,48]: to avoid agonistic interactions and successfully acquire resources, an individual must adjust their behaviour depending on the behaviour of the others. These cognitive demands are increased when living in large, complex groups, where not only current but also past behaviour of conspecifics is important; there is evidence that this pressure affects hippocampus morphology and perhaps structures in the neocortex [49]. Social play as juveniles may also be a key factor in learning to make behavioural adjustments, as individuals continually switch roles and behaviour patterns [47]. Housing with peers to allow play can have long-term effects on neural plasticity [50]. In general, animals reared in restrictive conditions, sometimes including sensory as well as social deprivation, seem to have difficulty with selective attention [51] and inhibitory control [52], suggesting atrophied or poorly developed neural systems (reviewed by [3]). Inhibitory control is key to performance in ‘re-learning’ tasks such as reversal learning, where animals need to withhold ‘go’ responses to the previously reinforced stimulus, while selective attention is needed for many types of cognitive task. Rodent studies have identified structures in the prefrontal cortex that are causally involved in different forms of behavioural inflexibility (see e.g. [32]), and neural pathways between the prefrontal cortex and the hippocampus play a role in the ability to inhibit behaviour [53]. It seems likely that these pathways are among those affected by social deprivation during development.

There are several features of social environments that may affect behaviour and cognition. For example, social housing often includes not only experiences of social interactions, including play, but also a broader range of physical stimuli, often including more space, of greater complexity. In the current experiments, for instance, the social complexity of the environment for Group calves, including both cows and calves, was by necessity accompanied by a larger and more complex physical environment. Pairs and individually housed calves had more directly comparable environments and did not differ in space available per calf, so their learning differences can more clearly be attributed to the social companion. Given that there was little difference in the learning performance of Group and Early Pair calves, it seems that learning was most affected by the presence of social companions rather than the other variables that differed in the Group setting (e.g. the physical environment, exposure to novelty, and provision of maternal care). This does not mean that those variables would never affect learning in calves, as there may have been a ceiling effect on performance here.

In terms of the range of stimuli provided, the Individual calves in this study were restricted from visual contact with other calves (mirroring the situation on many but not all farms using individual housing). Reduced visual stimulation can affect learning (reviewed by [3]). Having the opportunity to learn associations between visual cues and others, such as olfactory, that identify individual conspecifics could also be valuable during development. However, Gaillard and colleagues [6] found that 1- to 2-month old calves housed in pairs since birth made fewer errors in a reversal learning task than did individually reared calves that had visual contact with peers. These results suggest that providing visual contact is not sufficient to overcome the cognitive impairment. Similarly, other research on calf behaviour indicates that tactile rather than just auditory and visual contact with other calves is important for their welfare [54]. Finally, while the extensive interaction with humans in the current experiment provided a form of social contact and an opportunity for learning to respond to another living creature, human companionship is not believed to fully compensate for the presence of conspecifics (see e.g. [55]) and may not have the same effects on cognitive and social development.

The effects of social deprivation on learning are not necessarily permanent. Short-term isolation has been shown to have acute effects on brain function in piglets, where 15 min of social isolation altered corticosteroid receptor expression and reduced levels of mRNA that produce proteins necessary for neuronal development [9], and impaired learning performance under stressful conditions [56]. Even if the deficits are related to long-lasting changes to brain architecture, they may be reversible. Bredy and colleagues [57] demonstrated that the effects of low maternal care on cognition in rats could be reversed by environmental enrichment, but that the underlying differences in the brain remained. This is in contrast with studies of early deprivation in humans [58], which report lasting deficits or emotional problems. Partial recovery is possible in other primates [2,59]. The timing and severity of deprivation may also influence the extent to which recovery is possible.

Some individuals do not seem to develop cognitive deficits even when housed individually in early life, given that the average number of sessions to criterion in the reversal task did not differ between treatments when looking only at calves that successfully completed the task. This result might indicate that some individuals are generally better at coping with social deprivation, just as some individuals are more resilient to environmental stressors (e.g. [60]). Their superior learning performance might then be linked to better welfare within the Individual treatment. Alternatively, the difference in performance might result from differences in personality that affect this particular task but are unrelated to overall welfare or intelligence. For example, thigmotaxis (wall-hugging) in mice can affect performance in a water maze test, but these differences in performance likely have little to do with learning and memory [61]. In the present study, the ability to restrain approaches to the negative screen might relate to traits that are neutral for welfare or intelligence such as general locomotor activity, which affects the likelihood of the calf moving towards the screen before seeing the colour. Similarly, calves focused more on gaining reward than avoiding negatives such as punishment (cf. differences in regulatory focus in rats: [62]) might be more likely to make the error of persevering in a formerly correct response. While housing treatment might affect frequencies of the different personality types (see e.g. [63]), all calves of a given personality type might be equally successful regardless of their housing. These differences in learning might not generalize to other types of learning task requiring similar cognitive flexibility.

The mechanisms by which social housing affects learning still need further investigation: how does social interaction increase success if not through general anxiety or response to novelty? Is it simply through altered development of neural pathways specific to reversal and related forms of learning? If this is the case, the welfare effects of early social deprivation may be completely independent of the cognitive effects, and the latter may be quite difficult to reverse. Future research should investigate the effects of pairing calves at various ages, and repeat cognitive and behavioural testing later in life. The former would better test for sensitive periods and the latter would determine the duration of these effects, and thus their practical implications. For example, persistent cognitive deficits might reduce feed intake and juvenile growth rates, while on-going problems habituating to routine farm procedures such as breeding or milking, which might result from either the cognitive deficits or increased fear of novelty in individually reared calves, could reduce milk production and breeding success as well as making handling more difficult [64,65]. The partial success of the late pairing treatment, and the fact that the few Individual calves who were able to learn did so at the same rate as those from social housing, provides hope that, with some form of intervention, it is possible to overcome cognitive impairments associated with individual housing. Given the lack of a clear link between learning performance and fear-related behaviour in the tests used, however, interventions that address one problem will not necessarily resolve both.
